# A principles-based community health center for addressing refugee health: the New Canadians Health Centre

**DOI:** 10.3389/fpubh.2024.1440075

**Published:** 2024-11-13

**Authors:** Jessica Haight, Madeleine Kruth, Rebecca Gokiert, Augustine Botwe, Anja Dzunic-Wachilonga, Cristian Neves, Astrid Velasquez, Molly Whalen-Browne, Tehseen Ladha, Corinne Rogers

**Affiliations:** ^1^School of Public Health, University of Alberta, Edmonton, AB, Canada; ^2^Faculty of Medicine and Dentistry, University of Alberta, Edmonton, AB, Canada; ^3^New Canadians Health Centre, Edmonton, AB, Canada; ^4^Faculty of Education, University of Alberta, Edmonton, AB, Canada; ^5^Faculty of Nursing, University of Alberta, Edmonton, AB, Canada

**Keywords:** refugee, community health center, primary healthcare, model of care, Canada

## Abstract

The provision of timely and comprehensive healthcare is a fundamental aspect of resettlement for refugees, who often experience critical unmet health needs. In Canada, this includes connecting refugees with primary care providers to treat acute health conditions, as well as to provide longitudinal and preventative care. However, refugee access to healthcare is often complicated by numerous barriers, such as difficulty navigating health services, financial constraints, language barriers, discrimination, and limited access to physicians. Due to these unmet health needs and barriers to access, there has been a need for dedicated primary healthcare models for refugees that provide integrated and culturally safe care. In response, a refugee community health center, the New Canadians Health Centre (NCHC), was established in Edmonton, Alberta, Canada in 2021. The NCHC operates through an innovative, principles-based model that delivers interdisciplinary primary healthcare services to refugees that are culturally safe, include clients as partners in their care, and are grounded in principles of social justice, equity, and inclusion. Early and effective healthcare services are needed to address the health of refugees; however, there is a lack of research on the development and implementation of dedicated refugee health service models. This paper addresses this gap by providing an in-depth account of the opening of the NCHC as a principles-based community health center for refugees, including the process by which it was established in response to local needs and the development of its principles-based model for supporting refugee health. This community case description will support the development and implementation of other dedicated models for refugee health, and comes at a critical time in which there are rapidly growing refugee populations in Canada and internationally.

## 1 Introduction and context

As refugee numbers continue to increase globally, refugee health has been a topic of increased investigation in recent years (1). Many refugees have fled conflict and have experienced trauma, such as losing their homes and possessions, being separated from or losing family members, living in a refugee camp or temporary housing, and being survivors of violence (2). Due to exposure to traumatic circumstances and variable access to healthcare, refugees often struggle with undiagnosed mental and physical conditions for which treatment is critically needed (3).

Canada takes a leading role in refugee resettlement (1), resettling tens of thousands of refugees each year through the Refugee and Humanitarian Resettlement Program. Refugees are sponsored to come to Canada in three different classes, including government assisted (GAR), privately sponsored (PSR), or a combination of the two (blended visa office-referred refugees) (4). Additionally, refugee claimants can apply for refugee status from within Canada (4). Between 2016 and 2021, Canada welcomed 218,430 refugees as permanent residents (5), and over the next three years (2024–2026), Canada plans to settle another 220,000 (6).

Refugees receive healthcare insurance under the Interim Federal Health Program (IFHP), which provides access to physician and hospital services until refugees receive provincial or territorial healthcare insurance (4). While the IFHP provides refugees with healthcare insurance upon arrival, barriers can make it difficult for refugees to access needed care (2, 3). These barriers include challenges in service navigation, language, and financial and resource limitations (2, 3). Due to limited knowledge of the Canadian healthcare system and service eligibility under the IFHP, refugees are not always equipped to advocate for needed care (2). Financial constraints, unfamiliarity with transportation in Canada, and childcare responsibilities can make it difficult for refugees to travel to healthcare facilities (2, 7). Language barriers and cultural differences can impede patient-provider communication and treatment (7). Additionally, refugees may avoid healthcare services entirely due to past trauma and mistrust of institutions (2). Furthermore, evidence shows that family physicians can be hesitant to accept refugees as patients due to limited knowledge about refugee health, the complexity of refugee health challenges, the time intensive nature of medical visits, and language barriers (8). Due to these added barriers to access, there is a need for dedicated primary healthcare models for refugees that provide integrated and culturally safe care (3).

Dedicated refugee healthcare service models have been established in cities throughout Canada (9–11). However, prior to 2021, Edmonton was the only major city in Canada without a coordinated service model to address the health needs of refugees, despite being a designated receiving city for refugee settlement (12). In response to this gap, organizational leaders and researchers with experience supporting immigrants and refugees in Edmonton organized an advocacy group, the Refugee Health Coalition, which identified the need for a refugee health center. Through their collective efforts, they established the New Canadians Health Centre in 2021, a dedicated refugee community health center in Edmonton.

The NCHC operates through an innovative, principles-based model that delivers interdisciplinary primary healthcare services that are culturally safe, include clients as partners in their care, and are grounded in principles supporting social justice, equity, and inclusion. Services are delivered through an integrated and holistic approach, providing a continuity of care between family physicians and allied healthcare providers (e.g., nurses, counselors, health navigators). The NCHC serves GARs during their first two years in Canada, within which time the goal is to transition service users to a long-term primary care provider in their community.

Early and effective healthcare services are needed to address the health of refugees; however, there is a lack of research on the development and implementation of dedicated refugee healthcare service models (2), particularly in the Canadian context. Therefore, this paper addresses this gap by providing an in-depth account of the NCHC as a principles-based community health center for refugees in Edmonton, Canada. Specifically, we describe the process by which the NCHC was established in response to local needs and the development and characteristics of its principles-based model for supporting refugee health, with the goal that this research will support the establishment and implementation of other dedicated models for refugee health.

## 2 The establishment of the NCHC and its model

To inform the development of this community case description, a document analysis was conducted of materials from the NCHC which detail its creation and model characteristics (13). Specifically, documents that are foundational to the development and establishment of the NCHC were reviewed, including a review of interview findings previously reported by the NCHC in an internal report. These in-depth interviews were carried out between March and August 2022, soliciting the reflections of seven key informants who were significantly involved in establishing the NCHC, for the purpose of reporting on the journey of establishing the NCHC. Interview participants included members of the NCHC board, members of an NCHC partner agency, and others who were involved in the establishment of the NCHC in other capacities. Interviews took place in person or online, using the videochat platform Zoom, and were facilitated by two research assistants. Interviews lasted between 45 to 75 min and were audio-recorded and transcribed verbatim. Verbal and written informed consent were obtained from individuals to participate in interviews. These reported findings were reviewed to inform the development of this paper, and exemplar quotes from interviewees, referred to as “NCHC Partners,” are incorporated in the description below.

The examination of these documents and narratives was focused on gaining insight into the NCHC, and more specifically, (1) the establishment of the NCHC, which describes the context of refugee health in Edmonton and facilitators and barriers to its establishment, and (2) the NCHC’s current model of care, governance structure, approach to service provision, and research program. To ensure the credibility of this paper in accurately representing the context and experiences of the NCHC, member checking was also performed with NCHC Partners (15). Ethics approval was received from the University of Alberta Research Ethics Board (Pro00133232).

### 2.1 The process of establishing the NCHC

#### 2.1.1 The need to address refugee health in Edmonton

Edmonton is a large metropolitan city in Western Canada, with a population of approximately 1 million (6). With an estimated 49,110 refugees, Edmonton has one of the highest proportions of refugees *per capita* in Canada, making up 4.9% of the total population as of the 2021 census, and coming in behind only Mississauga (6.8%), Toronto (6.7%), and Ottawa (5.2%) (6). Despite being a designated receiving city for refugees, up until 2021, Edmonton was the only major city in Canada without a dedicated refugee health center. Although Edmonton had various settlement services and agencies committed to supporting refugees, it lacked a formal and sustainable model to address refugee health. As a result, refugees in Edmonton were at heightened risk of struggling to access health services and not receiving timely or adequate treatment. One NCHC Partner described this:

*When they [refugees] came here, they needed healthcare, but we did not have one real designated place or professionals that knew about refugee health… [so instead they go to] the emergency department. And you can get lost in the system so quickly.*

To address the provision of refugee health in Edmonton, a small-scale “New Canadians Clinic” (NCC) was opened from 2008 to 2017. The clinic was a product of several newcomer-serving community organizations that came together to address refugee health needs with funding from the provincial health agency Alberta Health Services (AHS). However, limited resources made it difficult for the clinic to sustainably respond to the health needs of refugees and it did not receive long-term funding to continue its work. After the NCC closed, AHS opened a small clinic to provide GARs with screening services; however, longitudinal care was not provided and services were insufficient to address refugee health needs in Edmonton. Therefore, following the closure of the NCC, community members in the refugee settlement sector in Edmonton recognized that resources and support were needed on a larger scale to effectively bring about change to address refugee health. In response, leaders in the refugee serving sector and healthcare providers launched a collaborative effort to address healthcare barriers faced by refugees and advocate for a new refugee health center. This led to the creation of the Edmonton Refugee Health Coalition (RHC) in 2017, which ultimately championed the development and establishment of the NCHC.

#### 2.1.2 A collaborative effort

The RHC is an advocacy group comprised of community members, healthcare providers, researchers, and academics, representing different newcomer-serving organizations and institutions in Edmonton. To advocate for a refugee community health center in Edmonton, the RHC developed a comprehensive strategy aimed at engaging community, decisionmakers, and provincial authorities.

A key component of this advocacy strategy was the collection and mobilization of evidence on refugee health needs and barriers and the role that dedicated health centers can play in meeting these needs through preventative care (3). This strategy was developed in response to an observed lack of awareness among decisionmakers on refugee health issues. Specifically, graduate students working with the RHC conducted and mobilized research on refugee health needs and the role of dedicated health centers through reviews of literature and reports on their findings (15, 16), targeting both policy makers and the academic community. This approach was a key tool in facilitating discussions with decisionmakers, as it provided credibility and improved decisionmakers’ knowledge of refugee health issues. One NCHC Partner explained:

*I think they [decisionmakers] started to realize how expensive it was to have all these [refugee] clients going to the emergency department, instead of actually giving them the tools to be able to navigate the health system and to access the right services [through a health center].*

In addition to collecting and mobilizing data, members of the RHC who worked with refugees shared their firsthand experiences of this work with decisionmakers, with the goal of providing narrative insight into the challenges associated with refugee health service provision in Edmonton. These testimonies from physicians, nurses, and other refugee-serving individuals helped to frame refugee health issues through real life experiences.

Members of the RHC mobilized knowledge on refugee health issues through letter writing campaigns, meeting requests, and presentations targeting decisionmakers and provincial authorities. NCHC Partners emphasized the importance of persistence, patience, and optimism throughout this process, particularly in the face of disappointments, such as efforts and conversations that did not result in support from decisionmakers. One NCHC Partner described this, *“We kept going, we did not give up. We kept talking to people and talking to people, going to different places.”*

A key milestone occurred when the RHC presented to the Edmonton City Council, after which the council agreed to support the RHC’s efforts to establish a refugee community health center. Specifically, the Edmonton City Mayor sent a letter to the Alberta Minister of Health in support of a refugee health center, which opened a door to a meeting with the health minister. These discussions with the health minister subsequently resulted in AHS providing funding for the RHC to conduct a formal community needs assessment, the purpose of which was to formally document the health needs of refugees in Edmonton and assess the type of service model that would be most appropriate for addressing these needs.

Community engagement became another key component of the RHC’s advocacy work. To compile the community needs assessment, the RHC engaged 154 participants across Edmonton. This included refugees with lived experience from 10 ethnocultural communities (*n* = 94), frontline social service and healthcare providers serving refugees (*n* = 58), and key informants (*n* = 2). Community members were asked about their health needs and services, as described by an NCHC Partner, *“We asked them, what does healthcare look like for you? What are your barriers with accessing healthcare? If you had an ideal model, what would it look like?”* A resulting report documented the health needs of refugees, barriers to healthcare in Edmonton, and key recommendations for effective health services, including the establishment of a dedicated refugee community health center (16).

Building on the findings from the report, members of the RHC then developed a formal Business Plan for the NCHC. This involved developing the mission, vision, and model for the NCHC, based on this engagement with refugees and healthcare providers, as well as a review of research evidence. The plan also involved formalizing partnership agreements with newcomer-serving community organizations that would contribute staff and resources to the NCHC (described next). Finally, physician leaders in refugee health secured a Clinical Alternative Relationship Plan with the government ministry Alberta Health in July 2021, which enabled physicians to work under an alternative funding model compatible with the provision of care for refugees. With funding for physician services in place and in-kind contributions from partner organizations to fund allied health practitioner roles, the NCHC was able to open.

**Figure 1:**
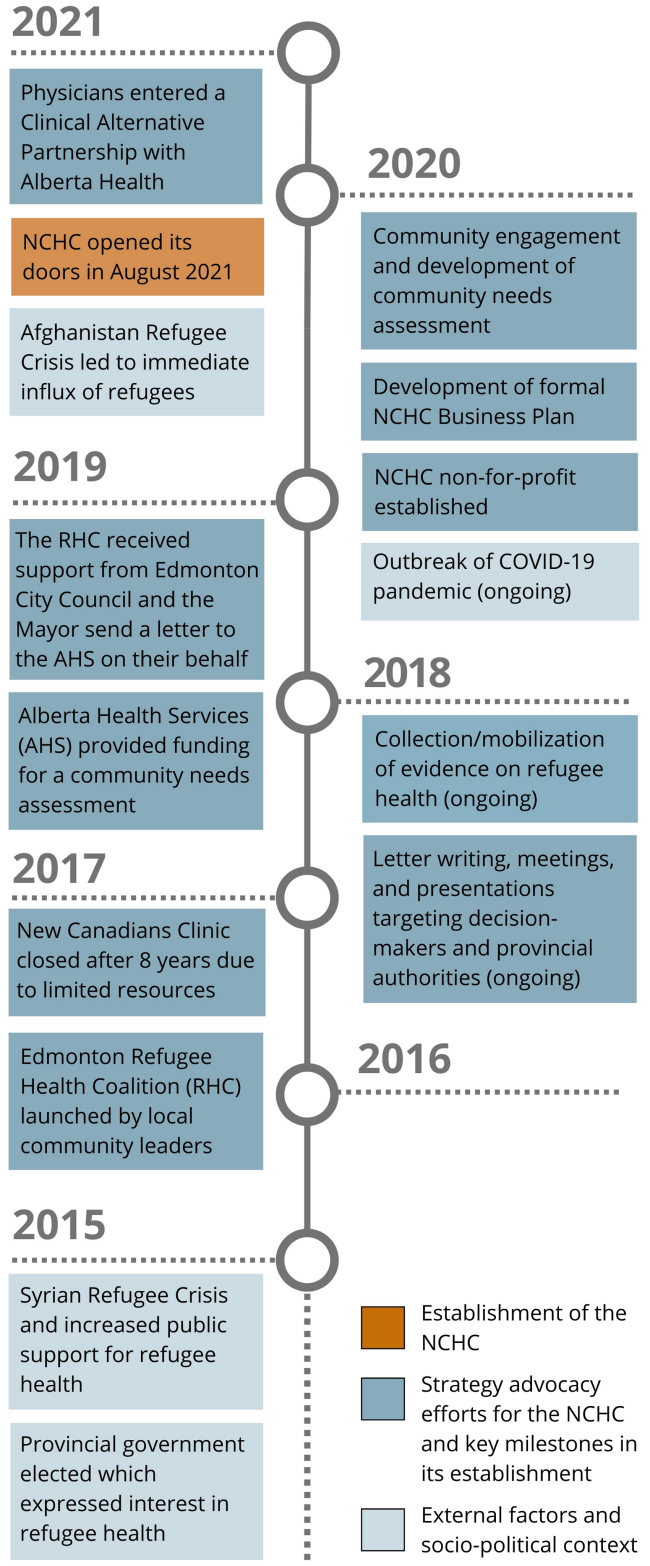
Timeline for establishing the NCHC.

Overall, the establishment of the NCHC was made possible due to the dedication of local champions for refugee health and a high level of commitment from community partner organizations in supporting these efforts. It should also be noted that social political context played a role in shaping the landscape for discussions around refugee health throughout this advocacy work. In 2015, the Syrian refugee crisis brought enhanced attention to the needs of refugees and members of the RHC observed increased support for refugee care in public spheres. At the same time, a transition in the provincial government led to more support for a refugee health center from decisionmakers. However, as of 2020, the onset of the COVID-19 pandemic created new barriers. Although the provincial government continued to support the RHC’s efforts to establish a health center, members of the RHC observed diminished public interest in refugee health due to increased socioeconomic hardship during the pandemic. The pandemic also posed a challenge for opening the clinic due to COVID measures and economic constraints in resources among agency partners. Despite these challenges, the NCHC persevered and successfully opened in August 2021 (the timeline to opening the NCHC is provided in Figure 1). There was a sudden and significant influx of refugees with a high level of need as soon as the NCHC opened its doors due to the Afghanistan refugee crisis in 2021. Therefore, the NCHC served approximately 500 clients within the first six months of operation (patients are referred to as “clients” by the NCHC to recognize their status as partners in their care).

### 2.2 The NCHC model

#### 2.2.1 The NCHC partnership

**Figure 2:**
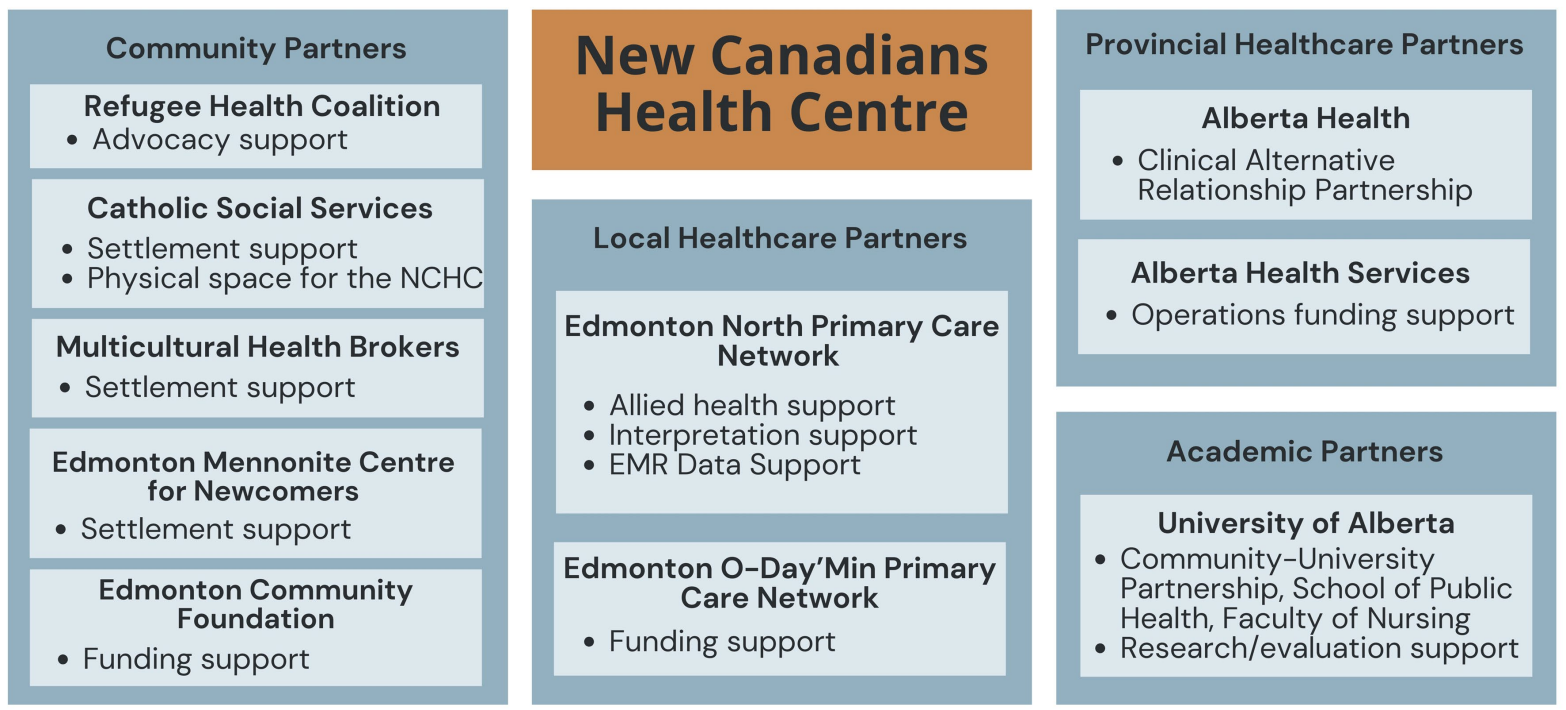
NCHC partnership.

Established as a not-for-profit, community health center, the NCHC represents a partnership between the RHC, AHS, Alberta Health, and other community, healthcare, and academic institutions, including Catholic Social Services (CSS), the Edmonton Mennonite Centre for Newcomers, the Multicultural Health Brokers Co-operative, the Edmonton Community Foundation, Edmonton North Primary Care Network, Edmonton O-Day’Min Primary Care Network, and the University of Alberta (see Figure 2). All partners have experience and expertise serving newcomers in Edmonton and contribute critical resources, funding, and operations support to the NCHC. One NCHC Partner commented, *“it takes a village to work a health centre.”*

Specifically, CSS provides intensive settlement supports for GARs during their first few weeks in Canada, including housing in their Reception House or other lodging, such as local hotels. CSS and the NCHC are also both located in the same building, which allows refugees to easily access NCHC services. Similarly, the Edmonton Mennonite Centre for Newcomers is a key settlement agency for newcomers. Clients can be referred to their settlement counselors, social workers, and language/cultural interpreters. The Multicultural Health Brokers Co-op also supports newcomers in Edmonton with cultural brokers and community support workers. Two local primary care networks (PCNs) also contribute vital resources to the NCHC, including the Edmonton North PCN, which provides the NCHC with allied health support (licensed practical nurse, nurse practitioner, behavioral health consultant), access to a phone-based interpretation service, and electronic medical records support, and the Edmonton O-Day’Min PCN provides the NCHC with some funding support. All partners are deeply committed to the goal of supporting refugee health through the NCHC principles-based model.

#### 2.2.2 Principles-based model

**Figure 3:**
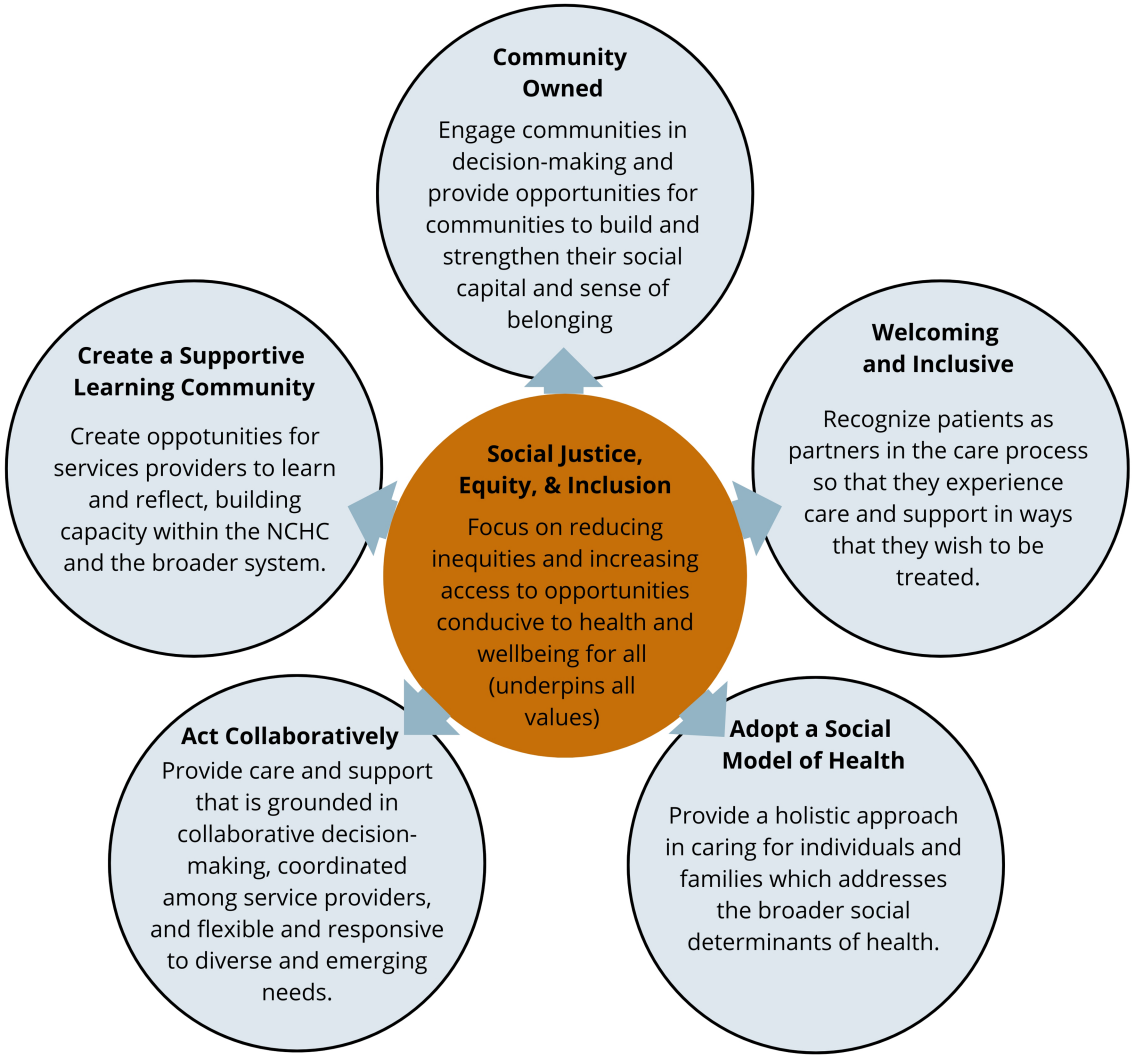
The NCHC principles.

The NCHC is a principles-based model, guided by core principles that support social justice, equity, and inclusion (see Figure 3). This is unique from other initiatives which may instead focus on specific outcome metrics, sometimes to the detriment of a shared culture of values and approach for achieving these outcomes (17). The principles-based model at the NCHC centers the mission of working toward the health and wellbeing of refugees at the forefront of all their work. The NCHC’s core principles were developed through a collaborative and iterative process, informed by the community needs assessment, input from refugees with lived experience and newcomer-serving health providers, and research literature. The five principles represent a living document and include the following:

1. Community owned: Engage communities in decision-making and provide opportunities for communities to build and strengthen their social capital and sense of belonging.
2. Welcoming and inclusive: Recognize clients as partners in their care process so that clients experience care and support in ways that they wish to be treated.
3. Adopt a social model of health: Provide a holistic approach in caring for individuals and families which addresses the broader social determinants of health.
4. Act collaboratively: Provide care and support that is grounded in collaborative decision-making, coordinated among service providers, and flexible and responsive to different and emerging needs.
5. Create a supportive learning environment: Create opportunities for service providers to learn and reflect, building capacity within the NCHC and the broader system.

All the work at the NCHC is grounded in these principles, ranging from high-level strategic decision-making to everyday client interactions. As one NCHC Partner described, *“You can see it [the principles] in everything. The way they [medical professionals] approach the clients, [and] the way they think about not just the health need or medication, but how is it going to be delivered?”* The following sections will outline how these principles are applied in practice through different aspects of the model.

#### 2.2.3 Governance structure

**Figure 4:**
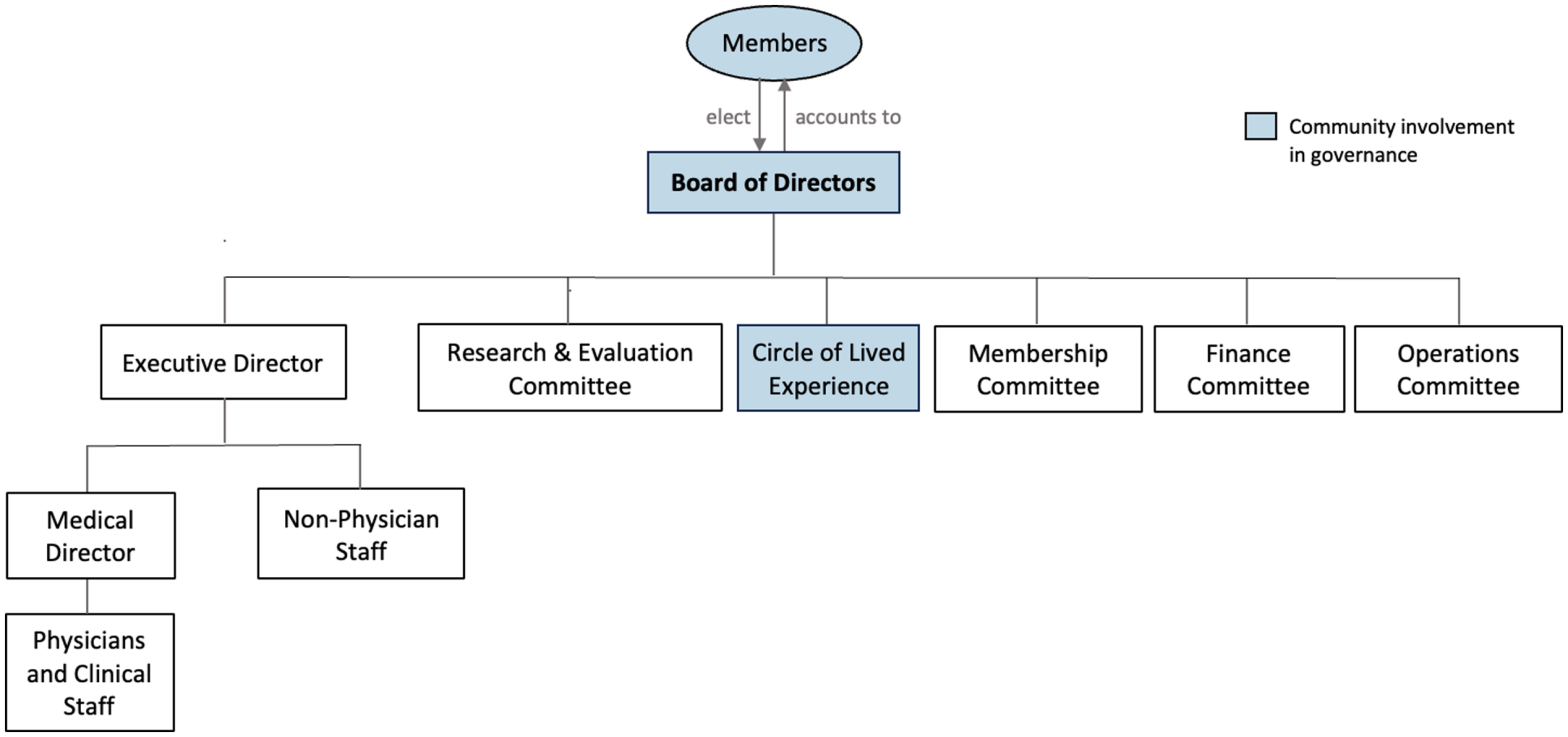
Governance structure.

The NCHC operates under an independent not-for-profit governance structure, with a Board of Directors serving as its main governance body (see Figure 4). The Board provides governance of the NCHC’s strategic directions and allocation of resources. The Board is elected by NCHC members during the Annual General Meetings or by other Board members and is, therefore, accountable to the community. NCHC members include diverse stakeholders, such as community members and leaders, refugees, medical professionals, and academics. This governance structure ensures that the NCHC meets its principle of being *community owned*. One NCHC Partner articulated this, *“[The NCHC is] informed and guided by community members who will actually benefit from the services of the Centre.”*

Under the direction of the Board, the executive director oversees all staff, and a medical director oversees clinical staff and physicians. There are also separate committees for membership, finances, operations, and research and evaluation at the NCHC. Importantly, the governance structure also includes representation of refugees, further supporting its principle of being *community owned*. Specifically, the NCHC has a Circle of Lived Experience, comprised of refugees who act as an advisory group and provide community perspective and feedback on the NCHC’s operations. This promotes a culturally safe, respectful, and compassionate approach, in line with the second principle for being *welcoming and inclusive*.

#### 2.2.4 Service provision

**Figure 5:**
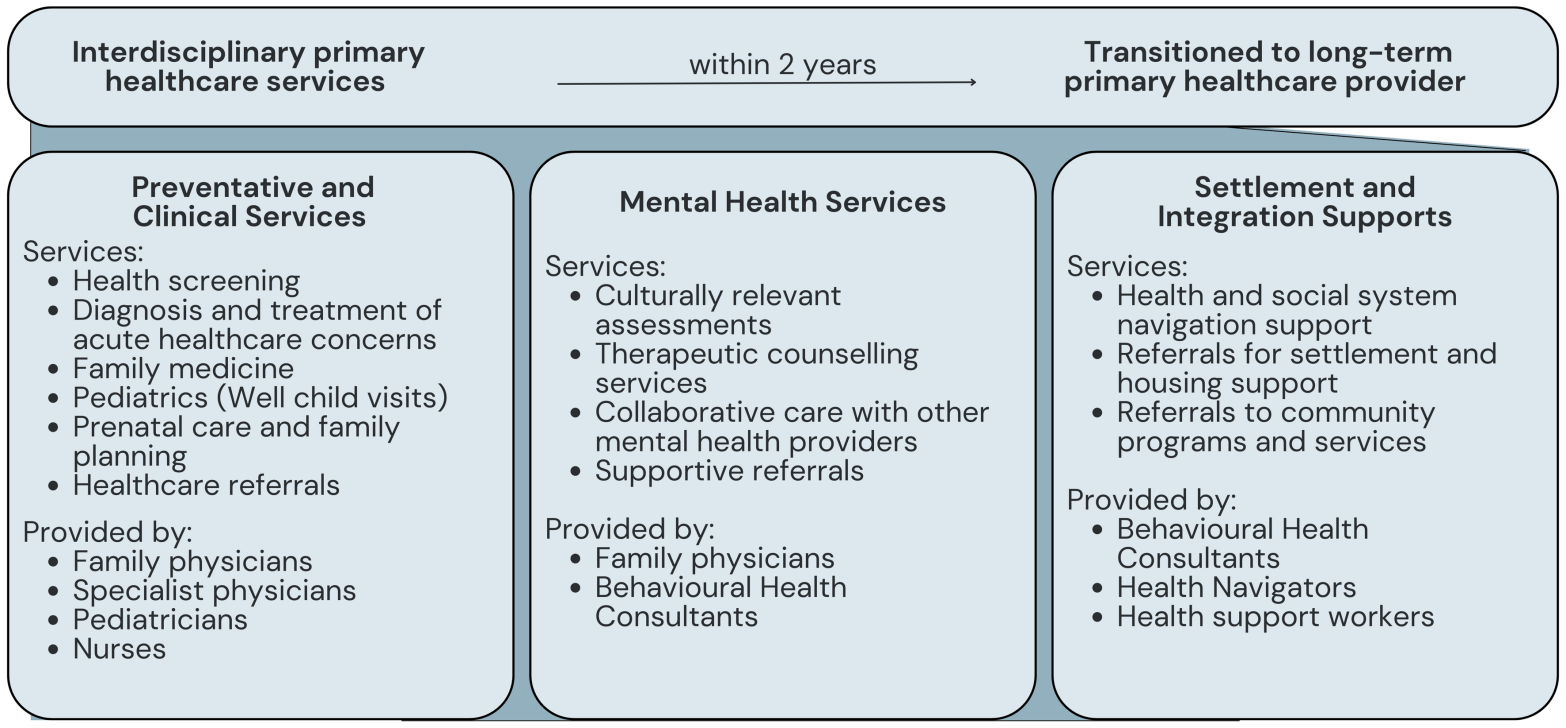
NCHC services.

The NCHC provides interdisciplinary healthcare services delivered by a collaborative team of physicians and allied health providers, in line with the fourth principle of *act collaboratively*. This interdisciplinary team provides a wide range of care, including preventative and clinical services, mental health services, and settlement and integration supports (see Figure 5). Following the third principle for a social model of health, the NCHC not only addresses refugees’ immediate clinical needs, but takes a holistic approach to health with the understanding that health is affected by physical, mental, and socioenvironmental factors, and that support is needed on these different levels for clients to achieve complete health and wellbeing (18). One NCHC Partner explained this:

*This is not a regular clinic where you go see your doctor, here is your prescription, go home…We need to make sure that you understand [what’s happening], you are referred to the right specialist, your mental health needs are addressed, and everything is taken care of.*

Specifically, the physicians working at the NCHC include six part-time family physicians, a part-time internal medicine physician, three part-time pediatricians, and a consulting infectious disease specialist. The allied health provider staff includes two part-time nurse practitioners, a part-time licensed practitioner nurse, a part-time behavioral health consultant, two health navigators, and three health support workers. The NCHC also has administrative staff, including a capacity-building coordinator, an operations manager, and medical office assistants. In addition to the in-house staff, the team also refers externally to a consulting psychologist and physiotherapist, who regularly see NCHC clients, and works closely with social workers, settlement counselors, cross-cultural counselors, and language/cultural interpreters from the settlement agency partners. One NCHC Partner commented, “*For one client, you have so many people working towards helping them…You have the doctor, the nurse, the health navigator, the community support worker, [and] counsellors. It’s many components that come together to make something like this happen.”*

In the delivery of services, clients are partners in their care and their active participation is encouraged in all aspects of healthcare decision making. Additionally, providers take a culturally safe approach to care, which involves recognizing that clients have unique health needs affected by sociocultural contexts, being responsive to these needs, and engaging clients in a respectful way that addresses power balances and creates a safe and supportive environment (19). One NCHC Partner explained, *“many of the clients come from parts of the world where they do not trust their health system. They do not trust doctors. There are awful things that have happened to them.”* Accordingly, providers *“need to understand how they arrived, under which circumstances, what are their needs, and how we can address those.”* To do so, the NCHC works with the settlement agency partners for joint learning on culturally safe care. Providing culturally safe care further speaks to the principle of fostering a *welcoming and safe environment.* One NCHC Partner explained this:

*It’s a safe environment…It’s a place where you come, and your problems and your needs are met because you can talk to somebody. And you know that somebody is actively working on resolving that issue for you or helping you to resolve that issue.*

Refugees are supported at the NCHC for their first two years in Canada. During this time, refugees receive comprehensive care that addresses their holistic health needs, including medical screening, treatment of acute health concerns, referrals to specialist care, and health navigation. As described by one NCHC Partner, “*We’re doing all this screening, we are taking care of the health needs, we are referring our clients to different specialists…and teaching the clients how to do things by themselves.”* While this provision of care is ongoing, the goal of the NCHC is to transfer clients to a long-term primary care provider in their community, in order to retain capacity to support incoming refugees. Since the NCHC opened its doors in September 2021, approximately 1,600 refugees have been served. At present, due to constraints in funding and capacity, the NCHC is only able to accept GARs; however, a future goal of the NCHC is to expand care to refugees of all status types.

#### 2.2.5 Research program

The NCHC formed a partnership with the Community-University Partnership for the Study of Children, Youth, and Families (CUP) at the University of Alberta to conduct research and evaluation on its principles-based model. Specifically, to guide research and evaluation activities, the NCHC Research and Evaluation Committee (REC) was created, comprised of community partners, clinical staff, allied health providers, and interdisciplinary academics and graduate and medical students who are conducting research or practicums at the NCHC. The REC also actively engages with the Circle of Lived Experience, to collaboratively set research priorities and plan research and evaluation activities.

The research at the NCHC is guided by a community-based participatory research (CBPR) approach, which involves the active engagement of those most closely impacted by the issue that is being researched across all phases of the research and its use (20). A CBPR approach allows for emergent design, equitably engages partners with diverse perspectives, and draws on the expertise of NCHC partners (20). This aligns with the principle of *act collaboratively* by actively involving NCHC partners in the research process. The research and evaluation of the NCHC is also guided by a principles-focused approach, which is focused on evaluating how, and to what extent, the project is addressing its guiding principles (18). The research and evaluation approach and partnership at the NCHC will be detailed in a forthcoming manuscript. Having research and evaluation embedded in the NCHC helps to achieve the fifth principle, *create a supportive learning environment*. Learnings identified in research and evaluation are actively incorporated into the NCHC practices on an ongoing basis.

## 3 Discussion

We have presented the model for the NCHC, a principles-based community health center with a specific mandate to support refugees, which delivers interdisciplinary primary healthcare services that are culturally safe, include clients as partners in their care, and are grounded in principles supporting social justice, equity, and inclusion. This paper addresses a lack of research on the development and implementation of refugee health service models by providing an in-depth account of the history, design, and structure of this innovative model for refugee health care.

The journey to establishing the NCHC required community collaboration, strategic advocacy efforts, and provincial support and funding. From the start, champions for the NCHC faced challenges, such as limited awareness of refugee health issues or support for refugees among decisionmakers and a lack of resources or funding. In response, the development and collective efforts of a large-scale partnership of local community and health partners were key to the establishment of the NCHC. The NCHC partnership engaged in multiple strategic advocacy efforts to garner support for a refugee health center, which involved mobilizing research evidence on refugee health and reaching out to decisionmakers and provincial authorities through letter writing campaigns, meeting requests, and presentations. These collaborative efforts led to relationship building and support from provincial decisionmakers (AHS and Alberta Health), which resulted in a funding model that enabled the NCHC to open in 2021. Furthermore, the NCHC’s community partners continue to contribute vital expertise, allied health services, and resources and funding to the which allows the NCHC to provide interdisciplinary allied health services that better address different upstream social determinants of health and support patients’ holistic health needs (21, 22).

The NCHC’s principles-based model (18), guided by core principles supporting social justice, equity, and inclusion, was informed by engagement with refugees on their health needs and an ideal model of services, through a community needs assessment (16). Rather than focusing only on specific outcome metrics, principles-based initiatives prioritize common values that guide their work and the processes by which they achieve outcomes (18). Principles-based approaches represent a growing area in the fields of systems change and evaluation, and are thought to be particularly responsive to dynamic environments and diverse populations due to their focus on culture and values (18). All the work at the NCHC is grounded in its foundational principles, from its structure and organization, its health service delivery, to its research and evaluation activities.

Specifically, the NCHC has a community governance structure with a Board of Directors, with representation of refugees through the Circle of Lived Experience, in line with the principle for being *community owned*. Community governance is an integral feature of community health centers (23); however, this level of community engagement, with representation of refugees, is unique in the available literature. From a health practice lens, the active participation of those with lived experience in the operation of services can help to make services more responsive and effective for their communities (24). Accordingly, this engagement supports the principle of making the NCHC *inclusive and welcoming* by incorporating input from the Circle of Lived Experience on culturally safe and appropriate services.

The NCHC provides interdisciplinary healthcare services delivered by a collaborative team of physicians and allied health professionals, which aligns with the principle of *acting collaboratively*. This allows the NCHC to provide a wide range of care, including preventative and clinical services, mental health care services, and settlement and integration supports, in line with the principle of a *social model of health*. An integral component of community health centers is the provision of comprehensive and integrated care (18). Research shows that dedicated refugee service models that offer integrated primary health care are associated with improved access to care and greater patient satisfaction (11, 25).

Notably, the NCHC also helps refugees to become integrated into the larger healthcare system. Specifically, the NCHC aims to have refugees transitioned to a long-term primary care physician in their community within two years of intake. The transition of refugees into care in their community is an important aspect of refugee care and has been the focus of “beacon” clinics, such as the Newcomer Health Clinic in Nova Scotia (9). Beacon clinics provide immediate medical care to refugees and then transition patients to local providers after six months (25). The NCHC has built relationships with community partners and local providers to support successful transitions to community care.

Finally, the NCHC also formed a research partnership with the University of Alberta (CUP) to evaluate their principles-based model and provide ongoing robust data related to their service provision. This CBPR program allows the NCHC to evaluate their model approach, the impacts of the model on refugee health, and actively incorporate findings into their practices and policies, supporting the principle of a *supportive learning environment*.

### 3.1 Methodological constraints

This community case description is specific to the establishment of the NCHC in Edmonton, Alberta; therefore, contextual factors will need to be considered when generalizing these experiences and learnings to other settings. However, due to limited literature on refugee health service models in Canada, this paper remains an important contribution.

## 4 Conclusion

Overall, the NCHC is an innovative principles-based community health center for refugees. This community case description provides unique insight into the development and implementation of a dedicated refugee health center in the Canadian context. Forthcoming manuscripts will evaluate the impact of the NCHC model on refugee health, incorporating the perspectives of refugees served by the center. More research is needed to guide accessible and effective health service models to foster refugee health.

## Data Availability

The datasets presented in this article are not readily available because data are restricted due to confidentiality agreements. Requests to access the datasets should be directed to Jessica Haight, jbhaight@ualberta.ca.
